# Ribodysgenesis: sudden genome instability in the yeast *Saccharomyces cerevisiae* arising from RNase H2 cleavage at genomic-embedded ribonucleotides

**DOI:** 10.1093/nar/gkac536

**Published:** 2022-06-24

**Authors:** Yang Sui, Anastasiya Epstein, Margaret Dominska, Dao-Qiong Zheng, Thomas D Petes, Hannah L Klein

**Affiliations:** State Key Laboratory of Motor Vehicle Biofuel Technology, Ocean College, Zhejiang University, Zhoushan 316021, China; Department of Molecular Genetics and Microbiology, Duke University School of Medicine, Durham, NC 27710, USA; Department of Biochemistry and Molecular Pharmacology, New York University Grossman School of Medicine, New York, NY 10016, USA; Department of Molecular Genetics and Microbiology, Duke University School of Medicine, Durham, NC 27710, USA; State Key Laboratory of Motor Vehicle Biofuel Technology, Ocean College, Zhejiang University, Zhoushan 316021, China; Hainan Institute of Zhejiang University, Sanya 572000, China; ZJU-Hangzhou Global Scientific and Technological Innovation Center, Hangzhou 311200, China; Department of Molecular Genetics and Microbiology, Duke University School of Medicine, Durham, NC 27710, USA; Department of Biochemistry and Molecular Pharmacology, New York University Grossman School of Medicine, New York, NY 10016, USA

## Abstract

Ribonucleotides can be incorporated into DNA during replication by the replicative DNA polymerases. These aberrant DNA subunits are efficiently recognized and removed by Ribonucleotide Excision Repair, which is initiated by the heterotrimeric enzyme RNase H2. While RNase H2 is essential in higher eukaryotes, the yeast *Saccharomyces cerevisiae* can survive without RNase H2 enzyme, although the genome undergoes mutation, recombination and other genome instability events at an increased rate. Although RNase H2 can be considered as a protector of the genome from the deleterious events that can ensue from recognition and removal of embedded ribonucleotides, under conditions of high ribonucleotide incorporation and retention in the genome in a RNase H2-negative strain, sudden introduction of active RNase H2 causes massive DNA breaks and genome instability in a condition which we term ‘ribodysgenesis’. The DNA breaks and genome instability arise solely from RNase H2 cleavage directed to the ribonucleotide-containing genome. Survivors of ribodysgenesis have massive loss of heterozygosity events stemming from recombinogenic lesions on the ribonucleotide-containing DNA, with increases of over 1000X from wild-type. DNA breaks are produced over one to two divisions and subsequently cells adapt to RNase H2 and ribonucleotides in the genome and grow with normal levels of genome instability.

## Graphical Abstract

**Graphical Abstract:**
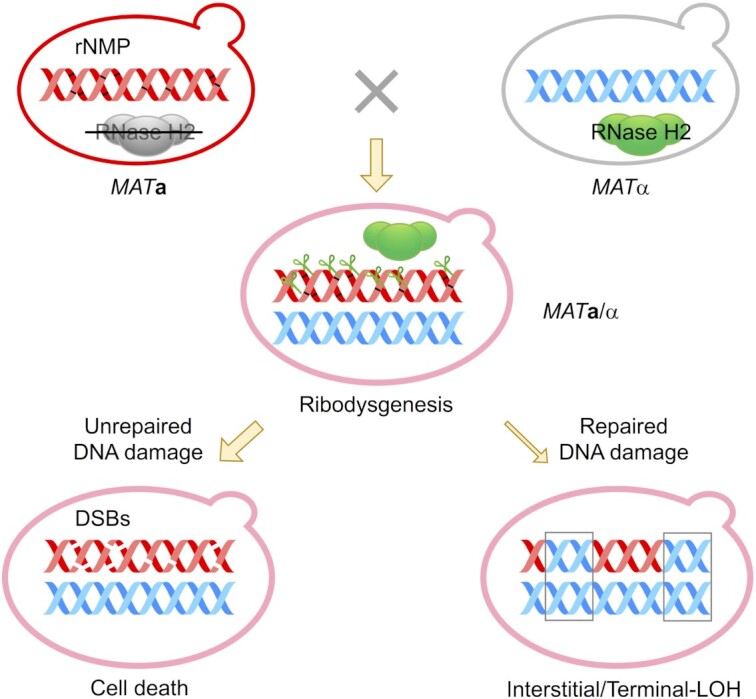
Massive pangenome instability and loss of heterozygosity (LOH) induced by RNase H2 cleavage at genomic-embedded ribonucleotides.

## INTRODUCTION

During genomic replication, the replicative polymerases incorporate ribonucleotides (rNTPs) instead of deoxyribonucleotides (dNTPs) at a significant rate, about 1 rNTP per 6500 dNTPs (1). These incorporated ribonucleotides maintain base pairing but, having the incorrect sugar for DNA, they cause helix distortion (2–8). All of the replicative DNA polymerases incorporate ribonucleotides but Pol epsilon (Pol2), contributes the greatest number of incorporated ribonucleotide residues during one replication cycle that are not removed during Okazaki fragment maturation (1,9,10).

The incorporated ribonucleotides are recognized by the enzyme RNase H2, a trimolecular complex of three different subunits. In the yeast *Saccharomyces cerevisiae* these are encoded by the *RNH201, RNH202* and *RNH203* genes. The RNase H2 enzyme initiates removal of the embedded ribonucleotides in a process termed Ribonucleotide Excision Repair or RER. Embedded ribonucleotides can be removed by topoisomerase 1 (Top1) or tolerated through bypass synthesis involving the replicative or translesion DNA polymerases (11–15). The Top1 removal pathway has preferential activity against ribonucleotides incorporated by Pol2 on the leading strand of DNA replication (15).

Variants of the replicative polymerases that incorporate increased levels of ribonucleotides have mutations near the active site or in the sugar discrimination pocket (16–18). The *pol3-L612G* allele was created through targeted mutagenesis of the conserved Leu^612^ residue in Motif A of the *Saccharomyces cerevisiae* Pol3 protein (19). It was identified as increasing spontaneous mutation rates (19) and later shown to also allow increased ribonucleotide incorporation, as demonstrated by the increased number of alkali-sensitive sites in genomic DNA in strains that are deficient in RER and carry the *pol3-L612G* allele (20).

Genome changes including single-base mutations, short insertions and deletions, inversions and translocations, aneuploidy and mitotic recombination events such as crossing over, gene conversion and break-induced replication (BIR) that lead to loss of heterozygosity (LOH), all contribute to shaping the genome of somatic cells by generating genetic diversity. While single locus or single chromosome assessment of spontaneous rates of these events are informative, recent advances have led to the ability to assess global genomic changes in diploid cells. In *S. cerevisiae*, use of diploid strains where the parental genomes differ by approximately 55 000 SNPs, the contribution of each parental genome and the detection of LOH can be determined without selection for any specific genetic event (21–24). Through mutation accumulation studies, global rates of spontaneous genomic alterations have been determined (22), providing a baseline for determination of specific effects of genome-destabililizing conditions of exogenous and endogenous damage. This approach provides a powerful genome-wide assessment of the impact of mutagenic conditions in terms of effects on rates and genomic locations of mutations and double-strand breaks (DSBs) that lead to genotypic changes in somatic cells.

Hybrid incompatibility is most often seen as sterility or lethality in interspecific hybrids (25) that results in gonadal failure, often due to nuclease activity of one parental genome against the other. One example is the mouse speciation gene *Hst1*, encoding the Prdm9 meiotic histone H3 methyltransferase (26), which confers sterility in intersubspecific hybrids due to male infertility. Other examples of hybrid incompatibility can result from negative epistasis, as has been found for variants of the mismatch repair (MMR) complex components in different *Saccharomyces cerevisiae* strains (27), resulting in an elevated mutation rate in hybrid diploids. In *Escherichia coli*, transfecting DNA containing mismatches and unmethylated DNA results in a failure of infectivity, from destruction of the transfecting DNA by the host MMR system (28). This mismatch-stimulated killing was found to be dependent on the RecA homologous recombination pathway, indicating that the killing reflected unrepaired DSBs.

Hybrid dysgenesis in *Drosophila melanogaster* results in hybrid sterility in females when the P transposable elements from one parent can destroy the germline genome of the other parent (29,30). Flies with P elements have the transposon in their genome and a P element repressor in the cytoplasm, to limit transposition, which occurs only in the germline. These are denoted as *P* strains. *M* strains have no P elements and hence no P element repressor. Of note in this example of hybrid dysgenesis is that the parental strains do not show reciprocity in crosses, that is, *P* × *P, M* × *M*, and *M* males × *P* females are fertile and produce viable offspring while *M* females × *P* males result in sterility as the cytoplasmic P element suppressor resides only in eggs, not sperm. In this cross, the P elements from the male parent are activated in and transpose within the germline cells of the progeny.

Here, we report a case of hybrid incompatibility in *S. cerevisiae* wherein parents with identical genotypes efficiently form viable homozygous diploids while parents in which one is wildtype while the other has high levels of incorporated ribonucleotides in DNA (reflecting a lack of RNase H2 activity and a mutation of DNA polymerase δ that incorporates more ribonucleotides than normal) results in a dysgenic cross with most zygotes failing to divide. We show that this is due to incision of the ribonucleotide-containing genome by the active RNase H2 of the wild-type parent, leading to recombinogenic DNA lesions.

## MATERIALS AND METHODS

### Strains and medium

All strains used in this study are listed in Supplementary Table S1. To examine the frequency of LOH events in zygotes that were heterozygous for *pol3-L612G* and homozygous for *rnh202*, we crossed the W303-1A-derived haploid LSY3454-2 (*rnh202::kanMX4 pol3-L612G*) with the YJM789-derived *rnh202::kanMX4 POL3* haploids MD893-1 or MD893-2. MD893-1 and MD893-2 are two independent transformants resulting from transformation of the *RNH202 POL3* haploid JSC20-1 (isogenic to YJM789 (31)) with a PCR fragment produced by amplifying genomic DNA of LSY3454-2 with the primers RH202 KOF (GCGATGAGAATTAAAGTC) and RH202 KOR (GCCCATTCGTTGGTAGTGAC). The resulting transformants have a replacement of *RNH202* with a *kanMX4* cassette. Two types of zygotes were produced, MD891 (cross of MD893-1 with LSY3454-2) and MD892 (cross of MD893-2 with LSY3454-2). Four zygotes of MD891 and two zygotes of MD892 were analyzed by microarrays. All experiments were performed growing yeast at 30°C on solid YPD medium or in liquid YPD medium.

### Imaging of zygote cells

Images of zygote cells after 4 days growth at 30°C were captured directly from the YPD plates using a Moticam 5+ camera placed in one ocular of an Aus Jena Zeiss dissecting microscope. Images were captured using the software Mitotic Images Plus 2.0 for OSX as Capture.bmp files.

### Alkaline gel electrophoresis and quantification of ribonucleotide density

Alkaline gel analysis was performed as described (32). Genomic DNA was isolated from yeast strains grown overnight in YPD using Qiagen 100/G tips (#10243) and Qiagen genomic DNA buffer set (#19060). 5μg purified DNA was incubated in 0.3 M KOH for 2 h at 55°C. Samples were mixed with 6× loading buffer (300 mM KOH, 6 mM EDTA, 18% Ficoll type 400, 0.15% bromocresol green, 0.25% xylene cyanol) and loaded onto a 1% agarose gel (UltraPure Agarose, Invitrogen #16500) made with alkaline buffer (50 mM NaOH, 1mM EDTA). Gels were run in 1X alkaline buffer (5 mM NaOH, 1mM EDTA) for 18 h at 30 V. Gels were neutralized by gently shaking in neutralizing buffer (1 M Tris–HCl, 1,5 M NaCl, pH 7.5) for 1 h and stained with 1× CYBR Gold.

Extraction and quantification of the gel data intensity profiles was carried out using MatLab code. The intensity profile (as a function of gel length) for each lane was calculated by averaging over the width of the lane followed by background subtraction. To obtain the background profile subtraction, the background profile was obtained by averaging the gap space on both sides of each lane. The Y-axis scale (in kb) was determined by extrapolating the specific peak locations of the different DNA fragments obtained in the intensity profile of the DNA ladder band, and this kb scale was transformed onto the Y-axis of the lane profiles. The maximum of each peak was normalized to 1 and the X-axis scale are arbitrary labels to 1.

### CHEF gel electrophoresis

5 ml yeast cultures were grown to saturation overnight at 30°C in liquid YPD. 2 ml of cells were used to make gel plugs. Cells were pelleted and washed twice with 1 ml CHEF TE (50 mM EDTA, 10 mM Tris pH 7.5) and resuspended in 150 μl CHEF TE with 4 μl zymolyase T100 (10 mg/ml) (USBiological #Z1004). Cells were immediately embedded in 1% agarose (Seaplaque GTG agarose, Lonza #50111) in 125 mM EDTA using BioRad plug molds (#1703713) and incubated in overnight at 37°C, followed by incubation in proteinase K (4 mg/ml) (Millipore-Sigma #70663). and 1% *N*-lauroyl-sarkosine overnight at 50°C. The plugs with washed with 1 ml CHEF TE three times for 60 min each. Plugs were run on a 1% agarose gel (Agarose-Certified Megabase Agarose Bio-Rad #1613108) in 0.5% TBE, using a Bio-Rad CHEF-DR II system, at 14°C at 6V/cm for 22 h with pulse times of 45/90. Gels were stained with ethidium bromide.

### Microarray analysis

The methods used for SNP-specific microarray analysis were described in St. Charles *et al.* (21). In brief, for about 13 000 SNPs that were heterozygous in the diploid strain used in our study, we designed 25-base oligonucleotides that were identical to the Watson and Crick strands of either the W303-1A-derived allele or the YJM789-derived allele. Since the yeast genome is about 12 Mb, the SNPs used in our study were located about 1 kb apart. These oligonucleotides were printed onto glass slides by Agilent Technologies. Sample preparations, hybridization protocols, and data analysis are described (21). DNA from the experimental strain was labelled with Cy5-dUTP and mixed with DNA from an isogenic control diploid strain labeled with Cy3-dUTP. Following hybridization to the glass slide with the oligonucleotides, the arrays were scanned with a GenePix scanner and GenePix Pro software. The normalized ratio of the median signal for each probe (635 nm/532 nm; *R*_M_) was obtained for each probe. These values were centered around a value of 1 by dividing the *R*_M_ of each probe by the average *R*_M_ for all probes.

### DNA sequencing analysis

From each colony derived from single zygotes, we generated a patch of cells and isolated DNA from the resulting patch without additional single-cell purifications. DNA was isolate using the Omega yeast DNA kit (Life Science Products) and was sequenced by the 150 bp-paired-end strategy using the Illumina NextSeq 500 platform. The data analysis was performed as described in Sui *et al.* (22).

### Analysis of single-colony isolates derived from zygote spore colonies

One important issue is whether the zygotes resulting from ribodysgenic crosses have on-going instability. Two arguments suggest that most of the LOH events are produced early after mating. First, the normalized coverage of most of the LOH events in the zygote colonies is 2-fold elevated for one allele and zero for the other allele, indicating that most or all of the cells in the colony derived from the zygote have identical patterns of LOH. Second, for two of the zygotes (HK10 and HK12), we purified single colonies (two per zygote) derived from the original zygote colony, and analyzed these isolates by microarrays. In HK10, of the 13 LOH events observed in the original zygote colony, 12 were present in the single-colony derivatives HK10A and HK10B; the LOH event on chromosome IV near 480 kb that was detectable by sequencing of the zygote colony and not in the microarray analysis of HK10A and HK10B involved a single-SNP that was not presented on the microarray. In addition, both HK10A and HK10B had T-LOH events on chromosome IV (at 725 kb in HK10A and 1.43 Mb in HK10B); these events were not evident in the sequencing data of the HK10 zygote because the cells contained a mixture of at least two types of cells. Both of the isolates derived from HK12 contained 11 of the 12 LOH events detected by sequencing; a small I-LOH event on chromosome XIV near coordinate 48 kb was not observed in the microarray data in either HK12A or HK12B because the SNPs diagnosed by sequencing were not represented on the microarray. Although HK12A had no additional LOH events relative to the sequenced HK12 isolate, HK12B had an I-LOH event on chromosome XI (breakpoint near 258 kb) and a T-LOH event on chromosome XII (breakpoint near 660 kb) that was not observed in the sequenced strain.

In summary, most of the LOH events in the zygotes appear to be produced immediately after zygote formation or at an early division following zygote formation. However, as discussed above, cells derived from a single zygote sometimes have differences in LOH patterns. These differences could arise as a consequence of the segregation of recombinant products produced in the cell cycle following zygote formation into different daughter cells. Alternatively, or in addition, DSBs produced prior to DNA replication could produce two broken chromatids that are repaired to produce LOH events with different breakpoints (21).

## RESULTS

### RNase H2 expression can cause lethality

**Figure 1.**
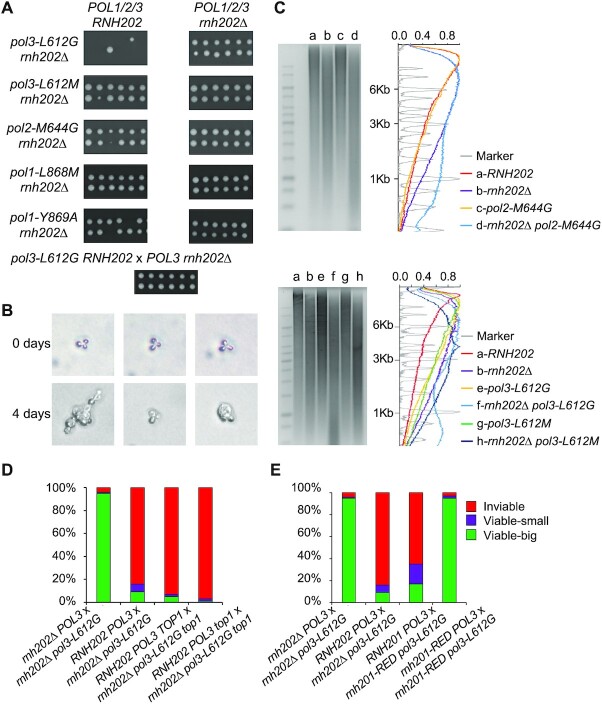
Lethality and genome rearrangements in the dysgenic cross *pol3-L612G rnh202Δ*x *POL3 RNH202*. (**A**) Twelve zygotes were pulled from each of the crosses shown. The top row shows the parental genotypes *POL1/2/3 RNH202* (wildtype) or *POL1/2/3 rnh202Δ*and the left column shows five polymerase mutant parents *pol3-612G, pol3-L612M, pol2-M644G, pol1-L868M, pol1-Y869A* in a *rnh202Δ*background. All crosses give twelve viable zygote colonies with the exception of *pol3-L612G rnh202Δ*x *POL3 RNH202*, and *pol1-Y869A rnh202Δ x POL1 RNH202*, which gave 10/12 viable diploids. The reciprocal cross *pol3-L612G RNH202* x *POL3 rnh202Δ*with the mutant alleles in trans gives twelve viable zygote colonies. (**B**) Microscopic examination of zygote colonies immediately following microdissection and after 4 days of incubation at 30°C. (**C**) Alkaline gel electrophoresis and analysis of genomic DNA. Genomic DNA was isolated form the indicated strains, treated with 0.3N KOH for 2 h at 55°C and run on 1% agarose gel in alkaline buffer. Gels were neutralized and stained with CYBR Gold. Densitometry tracings using MatLab of lanes in the alkaline gel. (**D**) Percentage of inviable, small viable zygote colonies and large viable zygote colonies from the indicated crosses involving *top1Δ*. 100 zygotes were micromanipulated for each cross. (**E**) Percentage of inviable, small viable zygote colonies and large viable zygote colonies from the indicated crosses involving *rnh201-RED*. 100 zygotes were micromanipulated for each cross.

We examined the ability of cells with DNA polymerase mutations that allow for increased incorporation of rNTP residues during replication to tolerate ribonucleotides in genomic DNA. Such strains include *rnh202Δ pol1-Y869A, rnh202Δ pol1-L868M, rnh202Δ pol2-M644G, rnh202Δ pol3-L612M* and *rnh202Δ pol3-L612G* (17,18,20). Although all these haploid strains are viable, the *rnh202Δ pol3*-*L612G*, when crossed to a wild-type haploid, had a unique phenotype, very poor zygote viability (Figure 1A). Similar lethality was seen in a cross of *RNH201 POL3* by *rnh201Δ pol3-L612G* (5 viable: 95 inviable for *rnh201Δ pol3-L612G* x *RNH201 POL3* versus 7 viable: 93 inviable for *rnh202Δ pol3-L612G* x *RNH202 POL3*), so the lethality is not restricted to *RNH202* genotype, but is a result of loss of RNase H2 function. In the *rnh202Δ pol3-L612G* x *RNH202 POL3* cross, between 10–20% of the zygotes were viable but often took several days of incubation before growth could be seen. The recovered colonies grew at different rates, as seen in Figure 1A. Microscopic examination of the zygotes that did not give rise to visible colonies showed that the zygotes did not divide or went through 3 divisions or less with grossly altered cells (Figure 1B). Growth inhibition was due to active RNase H2 enzyme provided by the wild-type parent as the cross *rnh202Δ pol3-L612G* x *rnh202Δ POL3* showed normal viability. This result suggests hybrid incompatibility of the two genomes as the result of the active RNase H2 enzyme from the wild-type parent acting on the embedded rNMP residues in the *rnh202Δ pol3-L612G* parent. As expected by this model, the reciprocal cross of *rnh202Δ POL3 x RNH202 pol3-L612G* did not show any inviability (bottom panel of Figure 1A). As the zygote viability was dependent on the parental genotype, we called this phenomenon ‘ribodysgenesis’. This property was not observed for any of the other DNA polymerase mutants that we examined.

To determine why only the *rnh202Δ pol3-L612G* showed ribodysgenesis, we used alkaline hydrolysis followed by gel electrophoresis to measure the amount of rNMPs in haploid strains of various genotypes; alkaline hydrolysis cleaves the DNA backbone at positions of incorporated rNMPs. As shown in Figure 1C, the *rnh202Δ pol3-L612G* strain genome was most sensitive to alkaline treatment of the strains we examined in this way, suggesting that a higher level of rNTP incorporation likely contributes to the hybrid incompatability. Published reports on the alkaline sensitivity of *rnh201Δ pol1-L868M* and *rnh201Δ pol1-Y869A* strains showed profiles that were not as shifted towards lower molecular weight as the *rnh202Δ pol3-L612G* strain (15,20). From these studies, we conclude that the *rnh202Δ pol3-L612G* has more rNMPs embedded in the genome than the other strains used in this study. Similar conclusions for the *rnh202Δ pol3-L612G* strain have been reported (33).

Embedded rNMPs that are not removed by RNase H2 and the RER pathway can be recognized and nicked by topoisomerase 1 (11,15,34). To determine whether Top1 cleavage had a role in the ribodysgenesis phenomenon, we used strains that were deleted for the *TOP1* gene in crosses. As seen in Figure 1D, the *TOP1* genotype had no effect of the zygote viability and a *rnh202Δ pol3-L612G top1Δ* x *RNH202 POL3 TOP1* cross had the same low viability as a *rnh202Δ pol3-L612G TOP1* x *RNH202 POL3 TOP1* cross (*P* = 0.05, Fisher exact test), showing that viability is not related to Top1 cleavage at embedded rNMPs. While loss of topoisomerase 1 activity in both strains in the *rnh202Δ pol3-L612G top1Δ* x *RNH202 POL3 top1Δ* cross gave even lower viability (*P* = 0.008, Fisher exact test), loss of topoisomerase 1 did not rescue viability.

RNase H2 recognizes and nicks at single rNMP residues in DNA as well as multiple contiguous rNMP residues and R-loops (35). The *rnh-201-RED* allele encodes an RNase H2 protein that cannot cleave single rNMP residues, but can remove multiple contiguous rNMP residues (35). Since the *rnh201-RED pol3-L612G* x *RNH201 POL3* cross resulted in zygote inviability whereas *rnh201-RED pol3-L612G* x *rnh201-RED POL3* resulted in normal zygote viability, the zygote lethality is primarily due to cleavage at single rNMP residues and not due to R-loop accumulation and processing (Figure 1E).

The few viable zygotes from a *rnh202Δ pol3-L612G* x *RNH202 POL3* cross showed significant evidence of DNA damage from DSBs or other recombinogenic DNA lesions by several criteria. First, we sporulated and dissected diploids from the viable zygotes and found evidence of chromosome loss and/or duplication, spore inviability, and diploids of mixed genotypes. An example of this latter is shown in Supplementary Figure S1A. Here both spore inviability and 2:2 segregation of colony size are readily visible. The genes *RNH202* and *POL3* are both located on chromosome 4, but on opposite arms. It can be seen in tetrad 1 both alleles for *RNH202* and *POL3* are segregating normally but, in tetrad 12, the *rnh202Δ pol3-L612G* chromosome has been lost and the *RNH202 POL3* chromosome has been duplicated. Second, CHEF (Clamped Homogeneous Electric Field) gel electrophoresis analysis of the zygote clones shows evidence of new chromosome bands that could come from translocations, and reduced intensity of bands that could come from chromosome loss or translocations (Supplementary Figure S1B); more definitive evidence for chromosome rearrangements will be presented below. Third, the *rnh202Δ pol3-L612G* strain requires functional Rad52 protein as the triple mutant *rnh202Δ pol3-L612G rad52Δ* was inviable, whereas the double mutant *pol3-L612G rad52Δ* is viable but slow-growing, indicating that even in a RNase H2-proficient background, significant DNA damage occurs (Supplementary Figure S2).

### Ribodysgenesis results in loss of heterozygosity (both interstitial and terminal)

The observation that some of the viable diploids from the *rnh202Δ pol3-L612G* x *RNH202 POL3* cross showed evidence of genome rearrangements prompted us to use assays that would allow capture of other rearrangements, including crossovers and BIR events between homologous chromosomes. We employed a genome-wide mapping approach using microarray analysis and whole-genome sequencing to examine a diploid that was heterozygous for approximately 55,000 single-nucleotide polymorphisms (SNPs) (21–24). This diploid was formed by crosses of haploids isogenic with W303-1A to haploids isogenic with YJM789 (21). For the experimental diploid zygotes, the W303-1A-derived haploid parental strain is *rnh202Δ pol3-L612G* (LSY3454-2) was mated to the YJM789-derived haploid parent (JSC20-1) which is *RNH202 POL3*.

As we observed with similar crosses described previously, with parental strains of these genotypes, most zygotes failed to divide. From two separate experiments, we isolated sets of six viable zygotes, HK1, HK2, HK4, HK7, HK9 and HK10 in one set and HK11-HK16 in the second. Unlike the mutation accumulation studies done in yeast diploids (36–39), we did not subculture strains over multiple generations. Instead, we analyzed the colonies resulting from growth of a single zygote (∼25 generations of growth) by using single-nucleotide polymorphism (SNP)-specific microarrays (21) or Illumina sequencing. As described below, this analysis allowed the detection of LOH events, as well as other types of genomic alterations. Since the results were very similar by the two approaches and since sequencing allows for the diagnosis of smaller alterations (e.g. single-base changes) that are undetectable using microarrays, our conclusions are based on the sequencing data.

**Figure 2.**
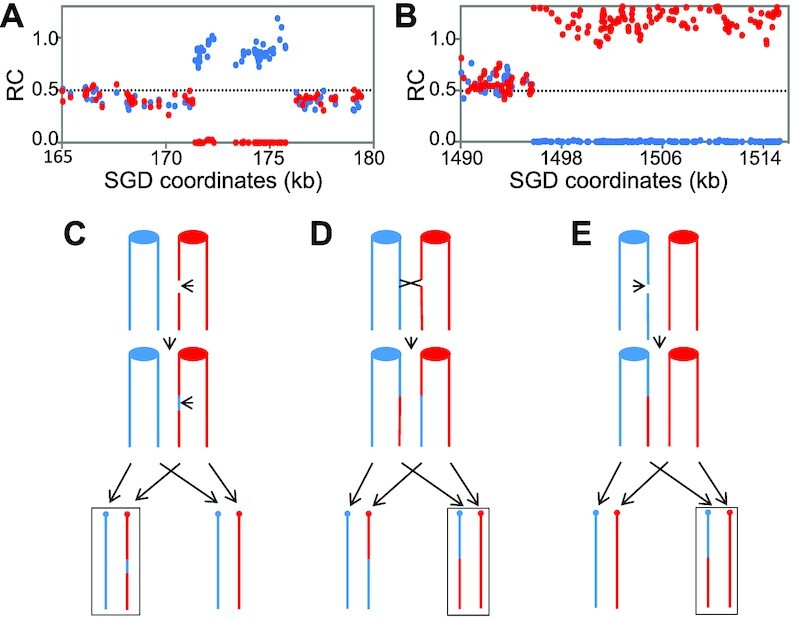
Depiction of I-LOH and T-LOH events. In (A) and (B), we show data from genomic sequencing experiments that diagnose interstitial (**A**) and terminal (**B**) LOH events. Each dot represents the ‘reads’ of allelic-specific single-nucleotide polymorphisms (SNP), with YJM789- and W303-1A-specific sequences indicated by blue and red dots, respectively. The ratio of coverage (RC) values on the Y-axis indicates the number of reads for the allele-specific SNP divided by the total number of reads for all of the SNPs in the genome. Thus, a value of 0.5 indicates heterozygosity, and values near 1.0 and 0 indicate two copies and no copies of the allele-specific SNP, respectively. The X-axis shows the chromosome coordinates as defined by the *Saccharomyces* Genome Database (SGD). (A) I-LOH event. This event involved the transfer of about 5 kb of sequences from one homolog to the other on chromosome IX in isolate HK2. (B) T-LOH event. This terminal LOH event was observed in isolate HK16 on chromosome IV. (**C**) Depiction of a gene conversion event resulting in I-LOH. The event is initiated by a double-strand break on the W303-1A-derived homolog (shown in red) followed by transfer of sequences from the YJM789-derived homolog. The ovals/circles show the centromere, and the arrows at the bottom of the figure indicate the pattern of chromosome segregation. The daughter cell with the pattern shown in (A) is outlined by a rectangle. (**D**) Generation of T-LOH by reciprocal crossing-over. A crossover between homologs, followed by segregation of the recombined chromatid with the unrecombined chromatid, can produce two daughter cells with reciprocal patterns of T-LOH. (**E**) Generation of T-LOH by break-induced replication (BIR). After a DSB in one chromatid, the resulting terminal fragment is lost. The centromere-associated fragment copies sequences from the other homolog, producing a T-LOH event in one daughter cell, but not the other.

DSBs in yeast cells are often repaired by homologous recombination between homologs or between sister-chromatids (40). Examples of the interstitial and terminal LOH events are shown in Figure 2A and B. Although we show the events initiated after the chromosomes have been duplicated, spontaneous events in wild-type cells are often initiated before DNA replication (21,41). As shown in Figure 2C, in an interstitial LOH event (I-LOH), the chromosome with the recombination-initiating DNA lesion is the recipient of information (42). I-LOH events, also termed ‘gene conversions,’ represent the non-reciprocal transfer of information from one homolog to the other. The amount of DNA transferred in crossover-associated conversion events during mitotic recombination is variable with a median size of about 10 kb in wild-type yeast cells (21,22). Conversion events unassociated with crossovers are shorter, about 3 kb (22).

In a T-LOH event, the region of LOH extends to the end of the chromosome (Figure 2B). Two pathways can produce T-LOH, reciprocal crossovers (RCOs) (Figure 2D) or break-induced replication (BIR) (Figure 2E). RCO events produce two cells with T-LOH. In BIR events, one broken chromosome fragment is lost, and sequences are duplicated by conservative DNA synthesis from the unbroken chromatid (42,43). In wild-type cells, RCO is more common than BIR (40).

DNA sequences from the twelve independent diploid isolates (six each from two experiments) were examined by whole-genome sequencing. The locations of the breakpoints for both I-LOH and T-LOH events in all strains are shown in Dataset S1-1, and schematic drawings of each of the LOH events are shown in Dataset S1-2. As we have noted in previous studies (21), whereas many of the I-LOH and T-LOH events involve simple patterns of LOH (e.g. Classes a1 and a2; Classes b1-b4 in Dataset S1-2), others involve multiple transitions between regions of heterozygosity and homozygosity or other complications. Since the complex events do not have a single unambiguous explanation, in the discussion below, we will emphasize the simple events.

The total number of LOH events observed in the zygotes was 120, about 10 events per zygote colony. Assuming that these events were accumulated in the 25 cell divisions required to form a colony, the rate of LOH was about 4 × 10^−1^/cell division. Since the rate of LOH events in an isogenic wild-type diploid is 4.6 × 10^−3^ (22), the zygotes have a rate of LOH that is elevated 87-fold above the wild-type rate. This estimate is likely a minimal estimate, since it is likely that most of the LOH events occurred in the first division after the isolation of the zygote or shortly thereafter. In support of this conclusion, when we sub-cloned two of the zygote colonies from a single cell to a colony and examined four such colonies by microarrays, very few additional LOH events were detected (described in Materials and Methods). Thus, the rate of LOH is likely elevated at least 2100-fold relative to the wild-type rate.

For the simple events, I-LOH exceeded T-LOH by a factor of about four; in an isogenic wild-type diploid, I-LOH was about three-fold more frequent than T-LOH (22). A critical point is that, in almost all (64 of 67) of the simple conversion events, sequences from the W303-1A-derived homolog were deleted and sequences from the YJM789-derived homolog were duplicated (data derived from Dataset S1). This result strongly argues that the recombination-initiating DNA lesions were on the homolog that contained the rNMPs (Figure 2C).

**Figure 3.**
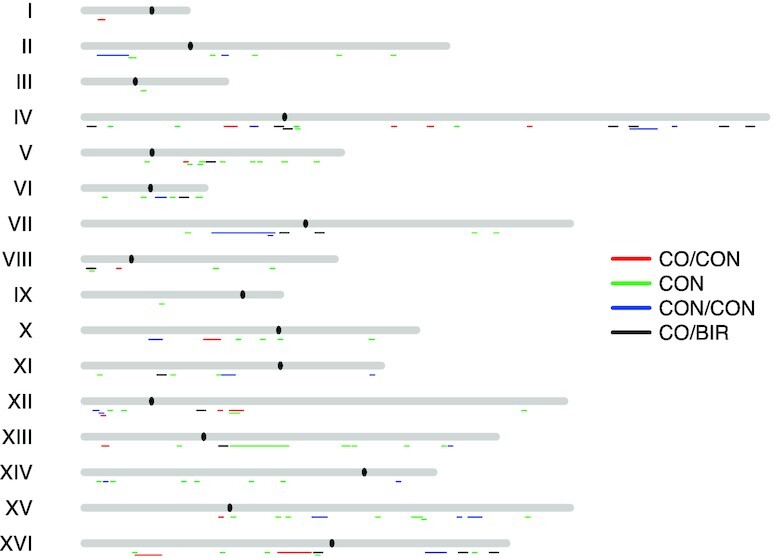
Distribution of LOH events. Based on the breakpoints of LOH events in HK isolates (Dataset S1), we mapped their positions along the 16 yeast homologs. The key to the colors showing the positions of the events is: red (CO/CON; T-LOH event associated with an I-LOH event); green (CON; simple I-LOH event), blue (CON/CON; complex I-LOH event), and black (CO/BIR; T-LOH event).

Both I-LOH and T-LOH events have transitions between heterozygous and homozygous regions that define the breakpoint of the event. These breakpoints presumably contain the location of the recombination-initiating DNA lesion (21). Using the method of defining breakpoints previously employed (21) (described in Materials and Methods), we mapped the breakpoints for both simple and complex LOH events (Dataset S1-1). The location of these events on the chromosomes is shown in Figure 3. The breakpoints are distributed widely over the chromosomes with no pronounced hotspots.

We also determined whether the breakpoints had over-representations of chromosome elements such as replication origins (ARS elements), transposable elements, tRNA genes, and a list of other elements (Dataset S2); the location and number of such elements are described in Zheng *et al.* (31). In our analysis of 18 such elements, most were not significantly enriched after we corrected for multiple comparisons. In the analysis of the breakpoints for both simple and complex events, there was an enrichment for the ‘tandemly repeated sequence’ category when we incorporated both simple and complex events (Dataset S2-1). Tandem repeats sequences are defined in the tandem-repeat-database (TRDB; https://tandem.bu.edu/cgi-bin/trdb/trdb.exe?taskid=0) (44). As in our previous study (45), we set the parameters for identifying tandem repeats as genomic regions with a minimal length of 24 bp that contained tandem repeats in the size range of 1–1998 bp. Since this same enrichment was not observed for the simple LOH events (Dataset S2-2), the significance of this relationship is not clear. If the LOH events are initiated by misincorporated rNMPs, the lack of a strong association with chromosomal elements is expected based on the genome-wide analysis of the distribution of rNMPs in yeast (20,46,47).

### Lack of LOH events in crosses of *rnh202Δ pol3-L612G* x *rnh202Δ POL3* strains

As described above (Figure 1E), zygote viability is high (>80%) in crosses of *rnh202Δ pol3-L612G* x *rnh202 POL3* haploid strains of the W303-1A genetic background. To examine the frequency of LOH in zygotes homozygous for the *rnh202* mutation, we mated the W303-1A-derived *rnh202Δ pol3-L612G* haploid to a haploid derived from YJM789 with the genotype *rnh202****Δ*** *POL3*. Six independent colonies derived from different zygotes were analyzed by SNP-specific microarrays. None of these diploids had LOH events, large deletions/duplications, or aneuploidy. This result supports the view that the poor viability in the *rnh202Δ pol3-L612G* x *RNH202 POL3* is likely the result of the high level of DSBs resulting from the RNase H2-catalyzed excision of ribonucleotides in the zygote.

### Aneuploidy and uniparental disomy in ribodysgenic crosses

We also detected five whole-chromosome alterations (Datasets 1–3 and 1–4). Four of these events were uniparental disomy (UPD) events, loss of one of the homologs followed by duplication of the other. Such events could represent two non-disjunction events: the first resulting in loss of one homolog in one cell cycle, and the second resulting in a gain of the remaining homolog in a second cell cycle. Alternatively, we showed previously that yeast cells, at low frequency (about 10^−7^ per homolog) undergo a meiosis I-like segregation pattern, producing two cells with reciprocal patterns of UPD; this process was termed ‘reciprocal UPD’ (48). Although our experiments do not unambiguously distinguish between these two mechanisms, by the first mechanism, we would expect high levels of monosomy and trisomy, which are not observed. The rate of UPD in wild-type cells was about 4 × 10^−6^/cell division, whereas the rate in the ribodysgenic isolates was 1.3 × 10^−2^/division if the UPD event occurred during formation of the zygote colony or 3.3 × 10^−1^, if the UPD event occurred in the first division after plating. Although these results suggest that ribodysgenesis may greatly elevate the frequency of UPD, this conclusion is based on a small number of events.

### Alterations in chromosome structure (large > 5 kb deletions/duplications and translocations)

Three of the twelve ribodysgenic diploids (HK13, HK14 and HK16) had large chromosome changes that could be detected by DNA sequence analysis and CHEF gel electrophoresis. In HK13, sequence analysis (Dataset S1-5) showed that chromosome XV has a heterozygous deletion of about 65 kb with breakpoints near SGD coordinates 600 kb (near a Watson-oriented Ty element) and 664 kb (near a pair of Watson-oriented delta elements [repeats associated with Ty elements]). By gel electrophoresis, we observed a novel band of about 1030 kb, slightly below the 1090 kb band representing the undeleted chromosome XV (Supplementary Figure S3A); this band hybridized to a probe derived from the *NDJ1* gene on chromosome XV (Supplementary Figure S3B), confirming the deletion.

Strain HK14 had a terminal deletion on the right arm of chromosome V (breakpoint near a Crick-oriented Ty at coordinate 446 kb) and a terminal duplication of the left arm of chromosome XV (breakpoint at a Watson-oriented Ty at coordinate 120 kb) (Dataset S1-5). As observed in our previous studies (31), this pattern of coupled terminal deletions and duplications is expected as a result of a translocation if the strain contains only one of the products of the translocation (Supplementary Figure S4); in addition, such translocations usually involve recombination between Ty elements or other dispersed repeats (31). Based on the location of the breakpoints of the deletion and duplication in HK14, we predicted that a recombination event between the two Ty elements at the breakpoints would produce a translocation of 560 kb that includes part of the left arm of XV and the right arm, centromere, and part of the left arm of V. We did not observe a novel chromosome of this size on the ethidium bromide-stained gel (Supplementary Figure S3A), presumably because this size is very similar to that expected from the native chromosome V (575 kb). However, a band of the expected size hybridizes to a probe derived from chromosome XV (*NDJ1*) (Supplementary Figure S3B), as well as to a probe near the predicted breakpoint of chromosome V (*GDI1*) (Supplementary Figure S5).

**Figure 4.**
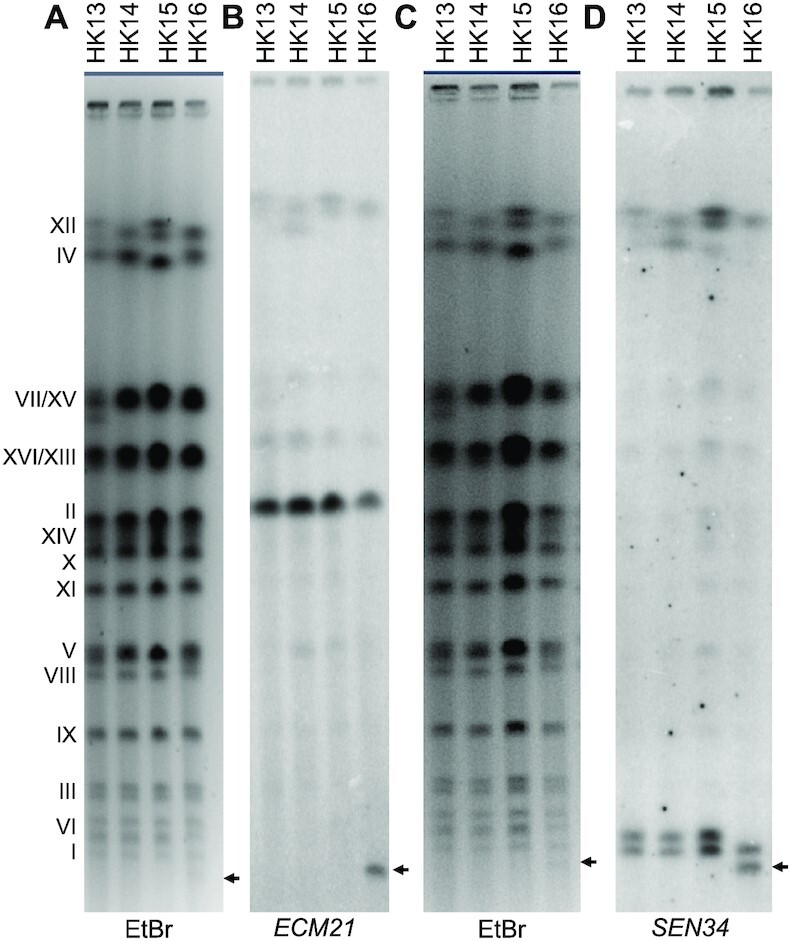
Analysis of a I-II translocation. As described in the text, based on the observations that isolate HK16 contained a terminal deletion on chromosome I and a terminal duplication on chromosome II with Ty elements at the breakpoints, we predicted a I-II translocation of 189 kb, slightly smaller than the smallest yeast chromosome (chromosome I). (**A**) CHEF gel analysis of four HK isolates. In the lane for HK16, we observe a faint band (marked with an arrow) at the expected position for the translocation. (**B**) Hybridization of a probe derived from chromosome II (*ECM21*) to the smallest band from the gel shown in (A); this probe also hybridizes to the position expected for the wild-type chromosome II. The bands near the top of the gel represent cross-hybridization of the probe to the ribosomal RNA genes on chromosome XII. This cross-hybridization is observed with many different probes. (**C**) Photograph of a different CHEF gel with the same HK isolates shown in (A). (**D**) Hybridization pattern for a probe derived from the end of chromosome I (*SEN34*). The two chromosome I homologs have slightly different sizes in the haploid parental strains, producing the two bands shown in HK13-15. In HK16, the larger form of chromosome I is lost because it forms part of the I-II translocation (the 189 kb band).

Strain HK16 had a terminal deletion on the right arm of chromosome I with a breakpoint near coordinate 160 kb (the location of a Crick-oriented Ty1), and a terminal duplication on the left arm of chromosome II with a breakpoint near coordinate 32 kb (the location of a Watson-oriented Ty2). Based on these breakpoints, one of the predicted translocations would be about 189 kb in size, slightly smaller than the intact chromosome I (230 kb). We observed a faint band at the appropriate position (Figure 4A and C) that hybridized to probes near the expected breakpoints (*SEN34* at coordinates 158–160 kb on I and *ECM21* with coordinates 25–28 kb on II) (Figure 4B and D).

In addition to the chromosome changes described above, we observed that chromosome XII, the chromosome containing the 1 Mb tandem array of rRNA genes, had frequent changes in size. Similar alterations are observed in wild-type strains as well as in strains with mutations affecting DNA replication (49). We quantitated the number of rDNA genes per strain by measuring the sequence coverage of the rRNA genes relative to the coverage of single-copy genes (Dataset S1-7). Since the rRNA genes derived from the W303-1A and YJM789 backgrounds are distinguishable by their SNPs, the number of repeats on each homolog could be determined separately.

In the isogenic wild-type diploid, there were 123 W303-1A-derived rRNA genes and 72 YJM789-derived rRNA genes (22), totalling 195 repeats. Among the twelve isolates, three (HK9, HK10, and HK16) had a terminal LOH event proximal to the rRNA gene locus, leading to loss of one class of repeat (Dataset S1-7). Among the remaining nine zygotes, the total number of repeats (average of 221) varied widely (138–328), as did the homolog-specific repeats. Unlike yeast strains under replication stress in which the number of rRNA genes is reduced (31,50), the zygotes have both elevated and reduced amounts of rDNA. This variation is likely generated by homologous recombination: unequal sister-crossovers or gene conversions, single-strand annealing events, and/or intrachromatid deletion and insertion events (31).

### Single-base mutations and small (<100 bp) in/dels in diploids from ribodysgenic crosses

Among the 12 sequenced zygote isolates, we observed 285 single-base mutations and 33 small in/dels (Dataset S3). The distribution of the single-base mutations among the six classes of alterations is very significantly different (*P* = 10^−11^ by Fisher exact test) from that observed in isogenic wild-type diploids (22). The distributions of different classes of single-base mutations for the zygotes and for an isogenic wild-type strain are shown in Supplementary Figure S6. Most (29 of 33) of the in/dels are deletions of one to three bases. These deletions were not flanked by short direct repeats unlike those observed in strains with low levels of DNA polymerase δ that likely reflect DNA polymerase slippage (31,51). As discussed below, the small in/dels are likely the consequence of the removal of misincorporated rNTPs by topoisomerase 1.

The mutations in the zygotes have several potential sources. First, some or most of the single-base mutations were likely introduced into the *rnh202Δ pol3-L612G* haploid prior to zygote formation by misincorporation errors of the mutant Pol δ. Alternatively, mutations could also reflect RNase H2*-*independent error-prone repair of misincorporated rNMPs. Since *pol3-L612G* strains have a much stronger mutator phenotype than strains with the *rnh202* mutation (14,19), the first source of mutations is likely the more important. In addition, we observed a 71-fold increase (5.4 × 10^−6^) in mutations at the *CAN1* locus in the *pol3-L612G* W303-1A strain, compared to a 2-fold increase (1.5 × 10^−7^) in the *rnh202Δ* strain, which we previously reported (14). This high mutation rate was only slightly increased (*P* = 0.46) in the double mutant *pol3-L612G rnh202Δ* strain to 95-fold (7.2 × 10^−6^), showing that most mutations arise from base misincorporation in this strain and not from processing or bypassing of embedded rNMPs in an error-prone manner.

Since strains heterozygous for the *pol3* mutations have a mutator phenotype (52), it is possible that some of the observed mutations were generated during growth of the zygotes. However, this possibility can be excluded as an important source of mutations. Mutations that were a consequence of on-going mutagenesis in the zygotes would be located on both of the homologs derived from both W303-1A and YJM789. We examined whether the mutations in the zygotes were linked to a W303-1A- or YJM789-specific polymorphism; since we used Illumina sequencing in our experiments, the polymorphism would have to be within about 300 bp in order to observe linkage. Of the 285 single-base mutations, 138 were linked to a polymorphism; of these 138 mutations, 132 were on the W303-1A-derived homolog (*rnh202Δ pol3-L612G* background) and 6 were on the YJM789-derived homolog (*RNH202 POL3* background). The *pol3-L612G* mutant displays marked bias for incorporation of rNTPs after C (CrN) (47), we would expect to see the CrN signature of single-base mutations if the mutations were a direct or indirect consequence of the incorporation of ribonucleotides. When we examined the base located 5′ to the mutant base (Dataset S3-1), we found 185/570 = 32% with a 5′C. The expected percentage for a random association with a 5′ C, based on a genomic G + C content of 38%, is 35%, indicating that the observed mutations do not reflect mutagenesis induced by ribonucleotides (*P* = 0.20 by chi-square test).

Although we cannot definitively determine whether the mutations in the zygotes were present in the *rnh202Δ pol3-L612G* haploid before mating or were introduced immediately after zygote formation, several lines of evidence suggest that most of these mutations are a consequence of mutations induced prior to zygote formation. First, we sequenced one of the haploid *rnh202Δ pol3-L612G* parental strains (LSY3454-2) used in constructing the zygotes, and detected 39 mutations relative to the isogenic wild-type haploid (Dataset S3-1); in our analysis, these mutations were excluded from the mutations found in the zygotes. Second, of the 285 mutations observed in the zygotes, we detected 17 identical mutations; these likely represent mutations induced during growth of the *rnh202Δ pol3-L612G* haploid prior to mating. Therefore, we favor the explanation that most of the observed mutations were produced in the *rnh202Δ pol3-L612G* haploid before mating.

Although the source of the single-base mutations in the zygotes is likely to be misincorporation errors by the mutant DNA polymerase, the in/dels probably reflect a different mechanism. Removal of rNMPs by topoisomerase 1 results in a high frequency of short (2–5 bp) deletions (11). These deletions often occur in regions that contain repeated dinucleotides. For example, three hotspot regions for 2-bp deletions in the *CAN1* gene contained (AG)_4_, (AT)_2_, and (TC)_3_ (11). Similarly, of the 33 short in/dels in the zygotes, 21 were two-bp deletions in two or three repeat ‘runs’ of dinucleotides (Dataset S3-2). Thus, most of these mutations were likely generated by Top1-mediated removal of rNMPs, although we cannot rule out other modes of mutagenesis.

One unusual feature of the mutations detected in the haploid strain LSY3454-2 is the percentage of ‘reads’ of the mutant base. For the single-base changes (Dataset S3-1), the mutant base was detected in greater than 95% of the sequence reads, as expected for a mutation in a haploid. However, for many of the in/dels, the mutant base represented <70% of the reads. These in/dels likely reflect mutations that occurred during the growth of LSY3454-2 before sequencing, generating a mixture of genotypes in the haploid. In support of this conclusion, the zygotes often lacked these in/dels or were heterozygous for the in/del. For example, the in/del at position 255089 on chromosome II was supported by 34% of the reads in LSY3454-2 (Dataset S3-2). Based on analysis of the raw sequencing data, in five of the HK zygotes, this in/del was found in about 50% of the reads, as expected for a heterozygous mutation. This in/del was not observed in seven of the HK zygotes. The high frequency of mixed in/dels in LSY3454-2, and the absence of mixed reads for the single-nucleotide variants, can be explained if the in/dels are generated at high rates at a small number of loci, whereas the single base alterations are widely distributed throughout the genome, each individual alteration at low frequency. Evidence for strong hotspots for the distribution of in/dels in *rnh201* strains has been previously presented (11).

### Loss of *RNH202* in recovered diploids from ribodysgenic crosses

We observed that the ribodysgenesis phenomenon arose from functional RNase H2 being introduced into a nucleus with a genome replete with rNMPs incorporated due to the *pol3-L612G* allele in a strain lacking a functional RNase H2 complex. Moreover, the zygote lethality was eliminated when the mating parents were both defective in RNase H2 enzyme. Thus, it was possible that all of the viable diploids lost the wild-type *RNH202* allele. To test this possibility, we directly sequenced the *RNH202* alleles in 52 recovered diploids. Only one diploid had a *de novo RNH202* mutation. Three diploids had become homozygous for the *RNH202* allele through crossing over/BIR or chromosome loss and reduplication, while four diploids had become homozygous for the *rnh202Δ* allele through crossing over/BIR. Thus, there does not appear to be any strong selection for loss of RNase H2 activity in the recovered diploids. This result argues for the lethal events occurring within the first zygotic cell cycle and stochastic escape from the lethality. Once the diploid is established, unless the genomic rearrangements inhibit growth, diploid cells that are heterozygous *POL3/pol3-L612G* and any *RNH202* genotype are able to grow as the lethality/DSB inducing lesions will have been removed by the active RNase H2 or tolerated if RNase H2 activity is absent.

In the twelve HK zygotes examined by DNA sequencing, three (HK11-HK13) were homozygous for the *rnh202Δ* allele as the result of a crossover/BIR event on the right arm of IV. Since we observed 33 T-LOH events and since the distance between *CEN4* and *RNH202* represents about 5% of the yeast genome, we expect about two events among the twelve strains, an insignificant departure from the observed number (*P* = 0.46 by chi-square analysis). Therefore, there does not appear to be a strong selection for events that result in loss of heterozygosity at the *RNH202* locus.

**Figure 5.**
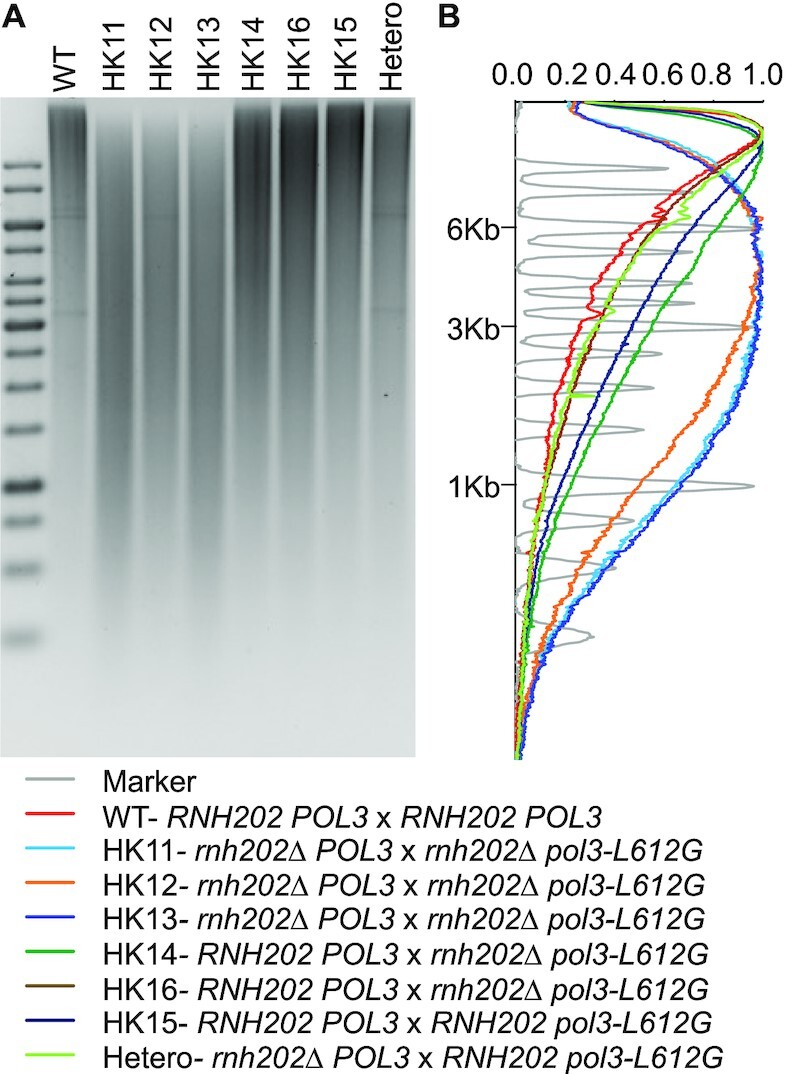
Alkaline gel analysis of diploids HK11-HK16. These diploids were formed by the cross *pol3-L612G rnh202Δ*x *POL3 RNH202*. Although all strains were expected to be heterozygous for the *rnh202Δ*mutation, strains HK11, HK12, and HK13 were homozygous for the *rnh202Δ*mutation and HK15 was homozygous for the wild-type allele. (**A**) Picture of alkaline gel with strains of different genotypes. The strains with the highest level of incorporated ribonucleotides are those that lack ribonuclease H2. (**B**) Quantitation of the size of the DNA fragments in the alkaline gels.

We also performed alkaline gel analysis on some of the recovered diploid isolates (Figure 5). Diploids that were fully wild-type (*RNH202 POL3/RNH202 POL3*), as expected, had the fewest misincorporated ribonucleotides, diploids of the *rnh202 POL3/rnh202 pol3-L612G* genotype had the most ribonucleotides, and diploids that were heterozygous for both the *rnh202* and *pol3-L612G* mutations had an intermediate level. These observations demonstrate that the *pol3-L612G* mutation is not completely recessive for the phenotype of ribonucleotide misincorporation. Indeed, a previous study on mutagenesis in diploids heterozygous *POL3/pol3-L612G* suggested that both alleles are active in diploids (52).

## DISCUSSION

Haploid strains with the *pol3-L612G* mutation have elevated levels of rNMP residues. In haploids with an additional mutation in genes encoding RNase H2, the level of rNMPs increases (Figure 1C). Nonetheless, haploid strains with both the *pol3-L612G* and the *rnh202* mutations have only a subtle effect on growth, resulting in an extended S/G2 (19,20). However, when RNase H2 is suddenly introduced into the rNMP-studded genome by mating to a wild-type strain, the *rnh202Δ pol3-L612G* strain, and not the other strains with mutant polymerases, has very poor zygote viability. The alkaline gel analysis shows that the *rnh202Δ pol3-L612G* strain has more alkali-sensitive sites in the genome than the other strains (Figure 1C), which could account for the sensitivity to a sudden burst of RNase H2 activity on the genome. We have shown that it is the presence of RNase H2 activity that is responsible for the zygote inviability. This conclusion is based on our observation of multiple chromosome rearrangements in the few surviving diploids (likely induced by DSBs) and the requirement for functional homologous recombination in the *rnh202Δ pol3-L612G* strain for viability (Supplementary Figure S2).

### DSBs and genomic rearrangements initiate on the rNMP-containing chromosomes

Using diploids where the parental chromosomes can be distinguished by 55 000 SNPs, we could determine the parental origin of the chromosomes that initiated LOH events. As shown in Figure 2A and C, the chromosome with the DSB is the recipient of information derived from the homolog. Thus, a duplication of sequences derived from the ‘blue’ chromosome (Figure 2A) is a consequence of the repair of a break on the ‘red’ chromosome (Figure 2C). Of note in our studies, it was not necessary to select for events or grow the diploids through many successive generations as is usually done in mutation accumulation studies. Instead, we allowed the zygotes to grow into colonies, which in itself took several days longer than normal. Every zygotic colony experienced multiple LOH events, with an increase of >1000-fold over wild-type, assuming that the LOH events were generated in the first division following zygote formation. Almost all simple interstitial events (64 of 67) were initiated on the rNMP-containing chromosomes and occurred within the first cell cycle.

### Model for zygote lethality by ribodysgenesis

In the absence of RNase H2 and a functional RER pathway, ribonucleotides incorporated by DNA polymerase epsilon (Pol2), can be processed by DNA topoisomerase 1 (15,53) to generate DSBs (54). Therefore, we tested whether DNA topoisomerase I contributed to the ribodysgenesis phenomenon and find no evidence that *TOP1* genotype affected zygote viability. In contrast, RNase H2 activity is required for zygote lethality and we infer produces the DSBs that lead to the LOH events and the subsequent zygote lethality.

Indeed, Top1 processing of embedded rNMPs is specific to leading strand synthesis by DNA polymerase epsilon (15) and thus the rNMPs incorporated by DNA polymerase delta (Pol3), must be either tolerated or most likely removed by other pathways in the absence of RNase H2 and RER. As we have shown that the zygote lethality arises from RNase H2 activity, there must be something specific about the number of rNMP residues and their genomic location that makes them susceptible to generating DSBs.

**Figure 6.**
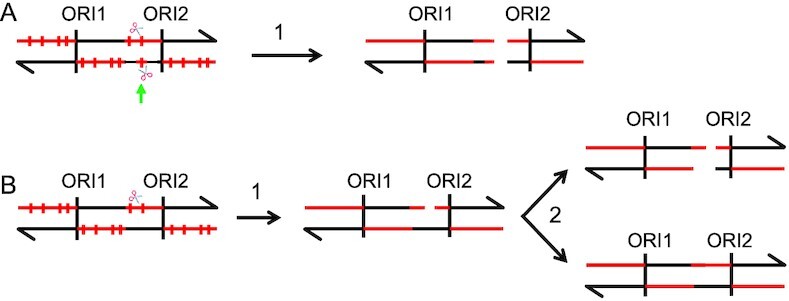
Two mechanisms for producing DSBs by RNase H2 cleavage of misincorporated ribonucleotides. In this figure, DNA molecules are shown as double-stranded molecules and ribonucleotides are depicted as red vertical lines. The density of ribonucleotides is expected to be much greater on the lagging strand during replication in the *pol3-L612G rnh202Δ*strain. (**A**) DSB produced by closely-placed nicks on opposing strands (shown by scissors). (**B**) DSB produced by replication of a nicked strand.

Recent studies have clarified the DNA polymerase usage during replication and in yeast leading strand is initiated by DNA polymerase alpha (Pol1) followed by a short region of synthesis by DNA polymerase delta (Pol3) for about 180 bp followed by a handoff to DNA polymerase epsilon (Pol2), which continues the processive leading strand synthesis (18,55,56). This replication pattern is universal for yeast origins based on genomic studies of polymerase usage (18). In Figure 6, we show the pattern expected for rNTP incorporation with respect to the origins. This figure also shows two mechanisms that could produce DSBs in the zygote. One mechanism is that RNase H2 creates closely opposed single-stranded nicks on opposite strands of the DNA.

Although most of the incorporated rNTPs would be expected to be located on the lagging strand, two conditions could introduce Pol delta-catalyzed mutations on both strands. First, it has been demonstrated that both strands of DNA are replicated by DNA polymerase delta (Pol3) for a short region near the origin. In a strain with the *pol3-L612G rnh202Δ* genotype, there is approximately one rNTP incorporated per 500 bp based on alkaline gel analysis (Figure 1); it is likely, therefore, that some origins will have rNMP residues on both strands. It is unlikely that this model explains our results, however, since in our analysis of the location of LOH breakpoints relative to various types of chromosome elements (Dataset S2), we did not find a significant association between breakpoints and origins. A related possibility is based on the observation that in yeast cells under replication stress, DNA polymerase delta can synthesize DNA on the leading strand as well as the lagging strand (56,57). In this scenario, excision of rNMPs closely opposed on opposite strands could produce a recombinogenic DSB (Figure 6A). A different mechanism is that the repair of very large numbers of misincorporated rNTPs following zygote formation may lead to nicked DNA molecules that, when replicated, produce DSBs (Figure 6B).

Regardless of the details of the process, we suggest that zygote lethality is a consequence of high levels of DSBs. Based on our observation of ten LOH events per zygote, it is likely that there are at least ten DSBs/genome. In addition, it is estimated that DSBs are repaired by sister-chromatid recombination about 20-fold more often than by inter-homolog recombination (40) and, therefore, the true number of DSBs induced by ribodysgenesis may be closer to 200 events/isolate. A dose of X-rays that produces about 250 DSBs/diploid yeast genome results in viability between 7–28% (58). This rough calculation is consistent with our hypothesis that the main cause of lethality in the ribodysgenic cross is DSBs resulting from the excision of ribonucleotides. One final question to be considered is why the removal of rNMPs in a haploid with the *pol3-L612G* mutation is not lethal in strains with the wild-type *RNH202* gene. One obvious possibility is that the density of rNMPs in a *pol3-L612G rnh202Δ* strain is considerably greater than in a *pol3-L612G* strain and, therefore, RNase H2 would be expected to produce more DSBs in the double mutant strain when RNase H2 is introduced by mating.

Our findings reported here on the presence of rNMPs in the genome leading to lethality when suddenly exposed to active RNase H2 may have some parallels to self-inflicted DNA damage by other nucleases. Caspase-activated DNase (CAD) has been shown to induce a wave of DNA damage in tumor cells to increase the level of DNA breaks such that tumors cells with high levels of damage from irradiation or chemotherapy are prevented from progressing into mitosis (59). We have shown that when a massive number of breaks are introduced into a genome by RNase H2, the escaped diploids have hugely increased (over 2000-fold) LOH which can also be accompanied by chromosome copy number changes. Indeed, LOH of essential genes is thought to lead to cancer vulnerabilities (60). While the genome instability we observe may be deleterious, it is possible that such a large number of changes could lead to a selective advantage for a subpopulation of cells.

Additionally, our findings may provide a general model for zygote death due to the accumulation of damage which could be of several types, including modified bases that are not ribonucleotides. Attack by the other genome could generate high levels of HR and genome instability and could relate to unknown causes of hybrid dysgenesis and infertility.

In summary, we have presented evidence that certain genotypes in yeast, when combined in a cross, result in very low zygote viability (dysgenesis). In our study, the dysgenesis was the result of a cross between one strain with a very high level of ribonucleotides and a second strain with the ability to excise these ribonucleotides, resulting in high levels of genetic instability. We term this event ‘ribodysgenesis.’ These findings may be relevant to understanding other examples of dysgenesis in yeast and in other eukaryotes. It is intriguing to consider that RNase H2 and ribonucleotides may underlie some cases of sudden genome catastrophe in tumor cells resulting in chromothripsis (61).

## DATA AVAILABILITY

The sequencing data are available in the National Center for Biotechnology Information (NCBI) Bioproject: Sequence Read Archive; Accession Number PRJNA314677.

## Supplementary Material

gkac536_Supplemental_Files
