# Supplementary material for: Ribodysgenesis: sudden genome instability in the yeast Saccharomyces cerevisiae arising from RNase H2 cleavage at genomic-embedded ribonucleotides
Source: Nucleic Acids Res. 2022 Jun 24;50(12):6890–902. doi: 10.1093/nar/gkac536 (PMC9262587; doi:10.1093/nar/gkac536)
Supplement: gkac536_Supplemental_Files [file gkac536_supplemental_files.zip › Supplemental file revised.pdf]

Supplementary Information for

Ribodysgenesis: sudden genome instability in the yeast *Saccharomyces cerevisiae* arising from RNase H2 cleavage at genomic-embedded ribonucleotides

Yang Sui[1,2], Anastasiya Epstein[3], Margaret Dominska[2], Dao-Qiong Zheng[1,4,5], Thomas D Petes[2], Hannah L Klein[3, *]

[1] State Key Laboratory of Motor Vehicle Biofuel Technology, Ocean College, Zhejiang University, Zhoushan, 316021, China
[2] Department of Molecular Genetics and Microbiology, Duke University School of Medicine, Durham, NC, 27710, USA
[3] Department of Biochemistry and Molecular Pharmacology, New York University Grossman School of Medicine, New York, NY, 10016, USA
[4] Hainan Institute of Zhejiang University, Sanya, 572000, China
[5] ZJU-Hangzhou Global Scientific and Technological Innovation Center, Hangzhou, 311200, China
* To whom correspondence should be addressed. Tel: (+1) 212-263-5778; Fax: (+1) 212-263-9484; Email: hannah.klein@nyulangone.org

This PDF file includes:

Tables S1

Figures S1 to S6

Legends for Datasets S1 to S3

Other supplementary materials for this manuscript include the following:

Datasets S1 to S3

Table S1. Yeast strains used in this study.

Strain	Genotype*	Source
HKY579-10A	*MAT* **a**	(1)
HKY2732-1D	*MAT* α	
HKY2190	*MAT* **a** *rnh202::KANMX4*	(2)
LSY3454-2	*MAT* **a** *rnh202::KANMX4 pol3-L612G lys2::NATMX4 ade3::GAL-HO*	(3)
HKY3363-6B	*MAT* α *rnh202::KANMX4 pol3-L612M leu2-ecoRI::URA3::leu2-bstEII*	
HKY2941-6C	*MAT* α *rnh202::KANMX4 pol2-M644G*	
HKY3458-7C	*MAT* α *rnh202::KANMX4 pol1-L868M*	
HKY3360-4B	*MAT* α *rnh202::KANMX4 pol1-Y869A*	
HKY3635-3A	*MAT* α *pol3-L612G*	
HKY2869	*MAT* **a** *pol2-M644G*	(2)
HKY3449-37-6C	*MAT* α *pol3-L612G*	
HKY3161	*MAT* α *pol3-L612M leu2-ecoRI::URA3::leu2-bstEII*	
HKY3525-5A	*MAT* α *rnh202::KANMX4 pol3-L612G top1::HPHMX4*	
HKY2459-1	*MAT* **a** *top1::HPHMX4*	
HKY3484-9D	*MAT* α *rnh201-P45D, Y219A pol3-L612G*	
HKY3279-6C	*MAT* **a** *rnh201-P45D, Y219A*	
HKY2352-19B	*MAT* α *rnh202::KANMX4 rad52::TRP1*	
HKY614-10B	*MAT* α *rad52::TRP1*	
HKY1093-5A	*MAT* **a** *CAN1 ADE2*	(2)
HKY2358-6A	*MAT* **a** *rnh202::KANMX4 CAN1*	
HKY2358-6B	*MAT* α *rnh202::KANMX4 CAN1*	
HKY3637-4C	*MAT* **a** *pol3-L612G CAN1*	
HKY3637-21C	*MAT* α *pol3-L612G CAN1*	
HKY3465-4A	*MAT* **a** *rnh202::KANMX4 pol3-L612G CAN1*	
HKY3465-29B	*MAT* **a** *rnh202::KANMX4 pol3-L612G CAN1*	
JSC20-1	*MAT* α *ade2-1 ura3 gal2 ho::hisG IV1510386::SUP4-o*	(4)
HK1, HK2, HK4, HK7, HK9-HK16**	*MAT* **a**/ *MAT* α *rnh202::KANMX/RNH202 pol3-L612G/POL3 lys2::NATMX4/LYS2 ade3::GAL-HO/ADE3 CAN1/CAN1 ade2-1/ade2-1 ura3-1/ura3 his3-11,15/HIS3 trp1-1/TRP1 IV1510386/IV1510386::SUP4-o ho/ho::hisG GAL2/gal2 RAD5/RAD5*	12 independent diploids from cross of JSC20-1 x LSY3454-2

MD893-1 and MD893-2	*MAT α ade2-1 ura3 gal2 ho::hisG IV1510386::SUP4-o rnh202::KANMX*	Isogenic independent isolates
MD891	*MAT* **a/** *MAT α rnh202::KANMX4/rnh202::KANMX pol3-L612G/POL3 lys2::NATMX4/LYS2 ade3::GAL-HO/ADE3 CAN1/CAN1 ade2-1/ade2-1 ura3-1/ura3 his3-11,15/HIS3 trp1-1/TRP1 IV1510386/IV1510386::SUP4-o ho/ho::hisG GAL2/gal2 RAD5/RAD5*	Cross of MD893-1 x LSY3454-2
MD892	*MAT* **a/** *MAT α rnh202::KANMX4/rnh202::KANMX pol3-L612G/POL3 lys2::NATMX4/LYS2 ade3::GAL-HO/ADE3 CAN1/CAN1 ade2-1/ade2-1 ura3-1/ura3 his3-11,15/HIS3 trp1-1/TRP1 IV1510386/IV1510386::SUP4-o ho/ho::hisG GAL2/gal2 RAD5/RAD5*	Cross of MD893-2 x LSY3454-2

*Most strains are of the W303 genotype *his3-11,15 leu2-3, 112 trp1-1 ade2-1 ura3-1 can1-100 RAD5*+ with the exception of strains JSC20-1 and derivatives described above. For W303-based strains only mating type and differences from the W303 genotype cited here are listed.

**Diploids HK9, HK11, HK12, HK13, and HK15 had a crossover between *CEN4* and the *RNH202* locus to homozygose the *RNH202* allele in the growing diploid. HK9, HK11, HK12, and HK13 are *rnh202::KANMX/rnh202::KANMX* while HK15 is *RNH202/RNH202*.

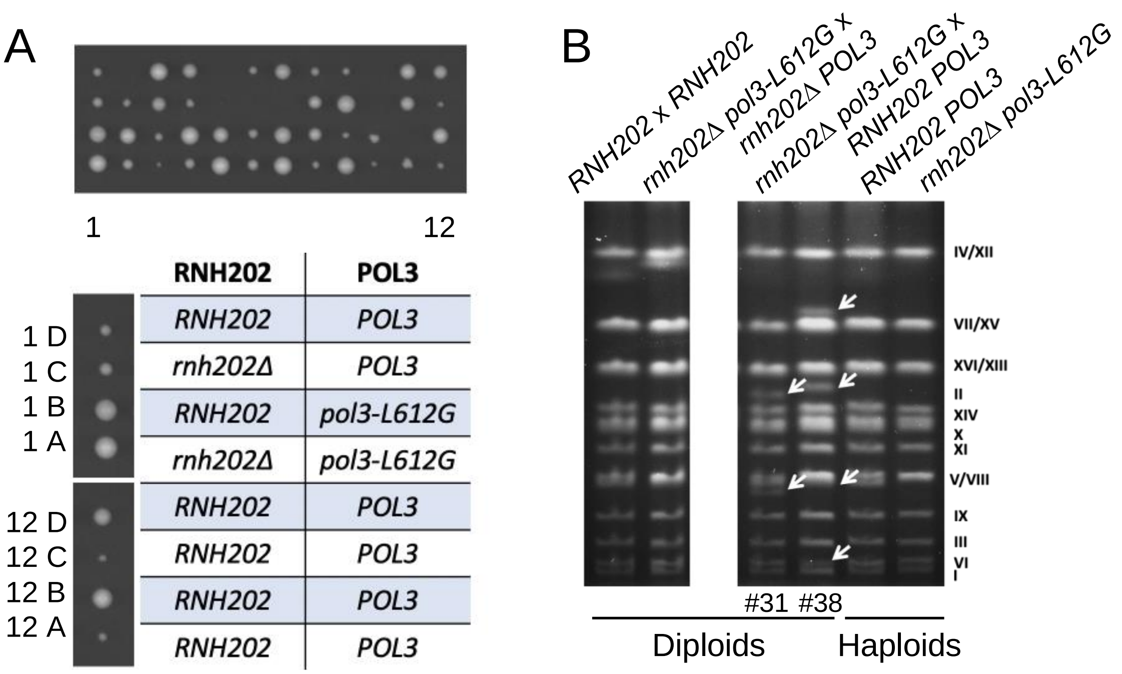

Figure S1. Meiotic segregation patterns and CHEF gel analysis of diploids derived from a ribodysgenic cross. A. Diploids derived from a ribodysgenic cross (*pol3-L612G rnh202Δ* x *POL3 RNH202*) were sporulated, dissected, and the segregation of the heterozygous markers was examined. The spore colonies were of various sizes, and the heterozygous markers did not always segregate 2:2. **B.** CHEF gel analysis of diploids from dysgenic crosses. Control diploids are shown on the left and the haploid parents are shown on the right. The numbers on the right of the gel indicate the sixteen yeast chromosomes. Two diploids, #31 and #38, are shown. The white arrows point to chromosomes of novel size or chromosomes of reduced intensity indicating chromosome loss.

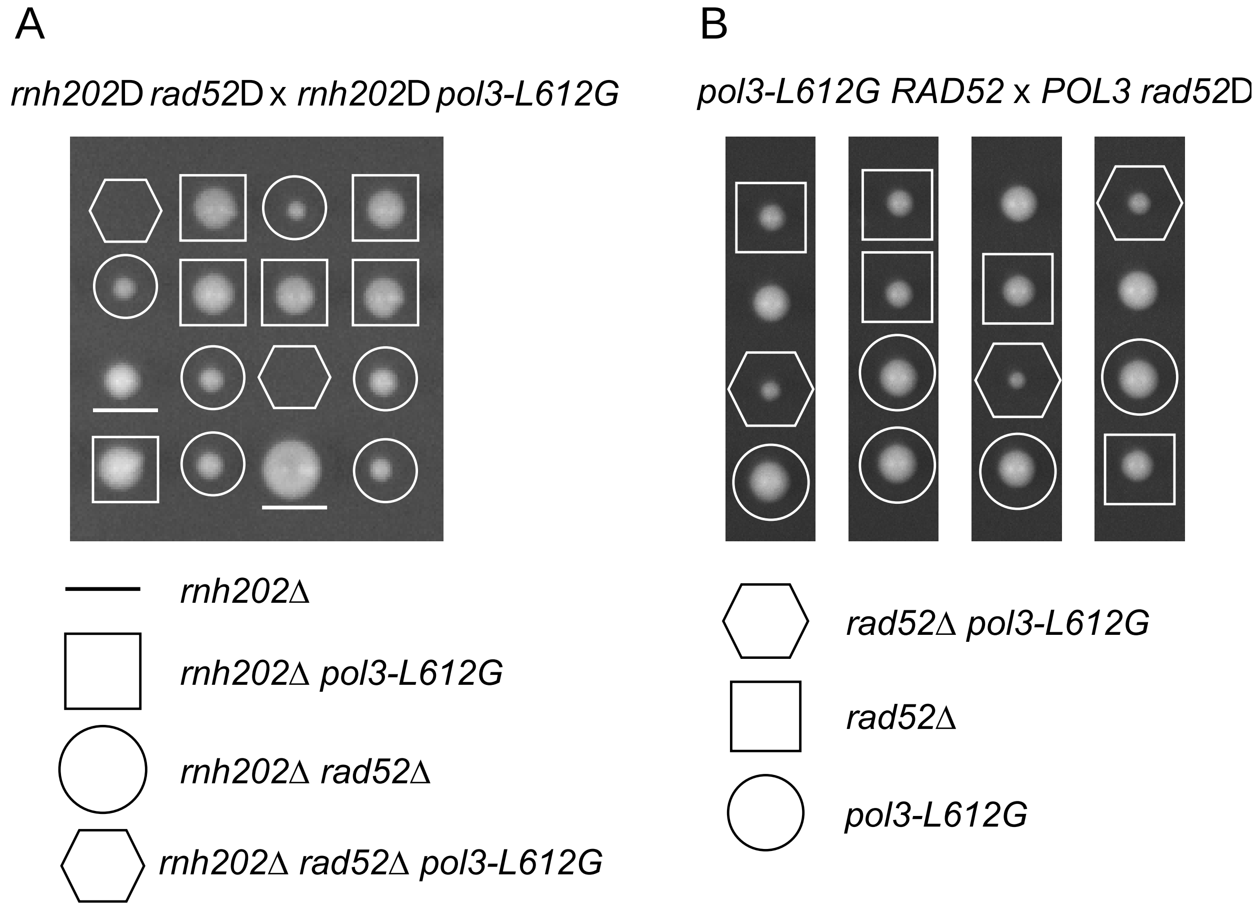

Figure S2. Requirement for *RAD52* for viability in *rnh202Δ pol3-L612G* haploid segregants. **A.** Tetrads, shown vertically, from a cross of *rnh202Δ rad52Δ* x *rnh202Δ pol3-L612G.* The hexagon symbols indicate spore segregants of the *rnh202Δ rad52Δ pol3-L612G* genotype, which are lethal. Note that the *rnh202Δ rad52Δ* genotype is viable. **B.** Tetrads, shown vertically, from a cross of *pol3-L612G RAD52* x *POL3 rad52Δ.* The hexagon symbols indicate spore segregants of the *rad52Δ pol3-L612G* genotype, which are viable but slower growin

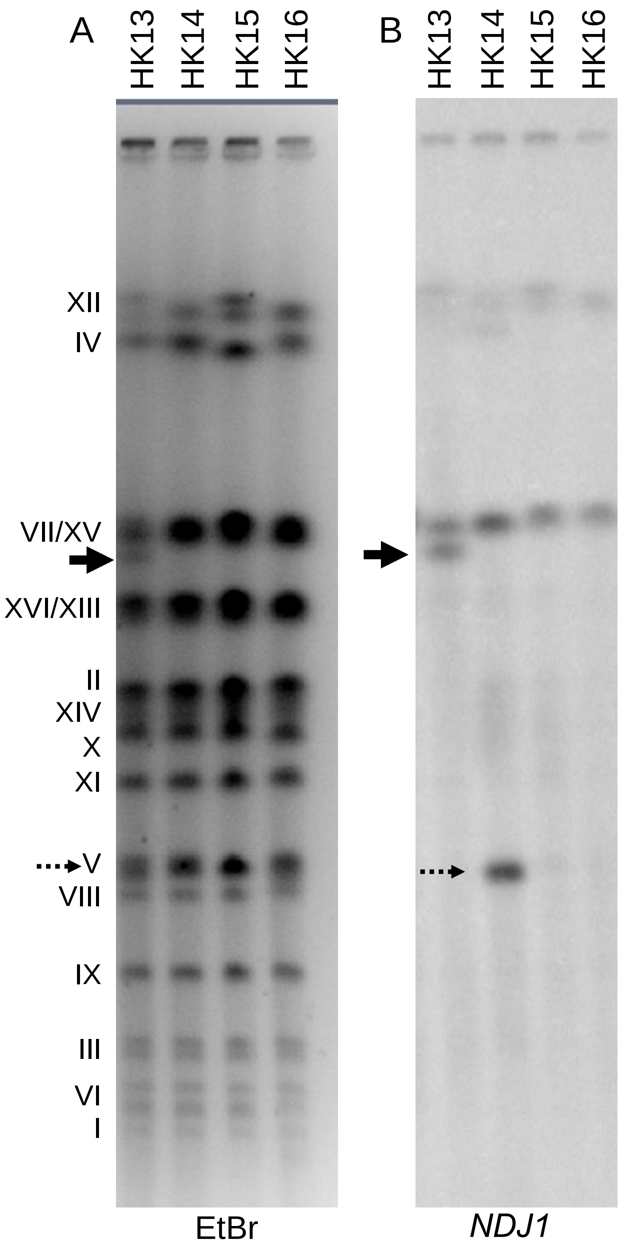

Figure S3. Analysis of a large deletion by CHEF and Southern analysis. By sequence analysis, isolate HK13 had a heterozygous deletion of about 65 kb on chromosome XV. **A.** EtBr staining of a CHEF gel containing samples from isolates HK13-HK16. In the lane with HK13 DNA, there is a band (marked with a solid arrow) that is about 65 kb shorter than chromosome XV. **B.** Southern analysis of the samples shown in Fig. S3A. A filter derived from the gel was hybridized to a chromosome XV-specific probe (*NDJ1*). This probe labeled two bands in the HK13 lane, one representing the intact chromosome XV and the other (marked with a solid arrow) representing the deletion derivative of XV. As shown in the HK14 lane, this isolate has two chromosomes that hybridize to *NDJ1*, the intact chromosome and a chromosome that is approximately the same size as chromosome V (560 kb). As discussed in the main text and Fig. S3, this chromosome is a V-XV translocation.

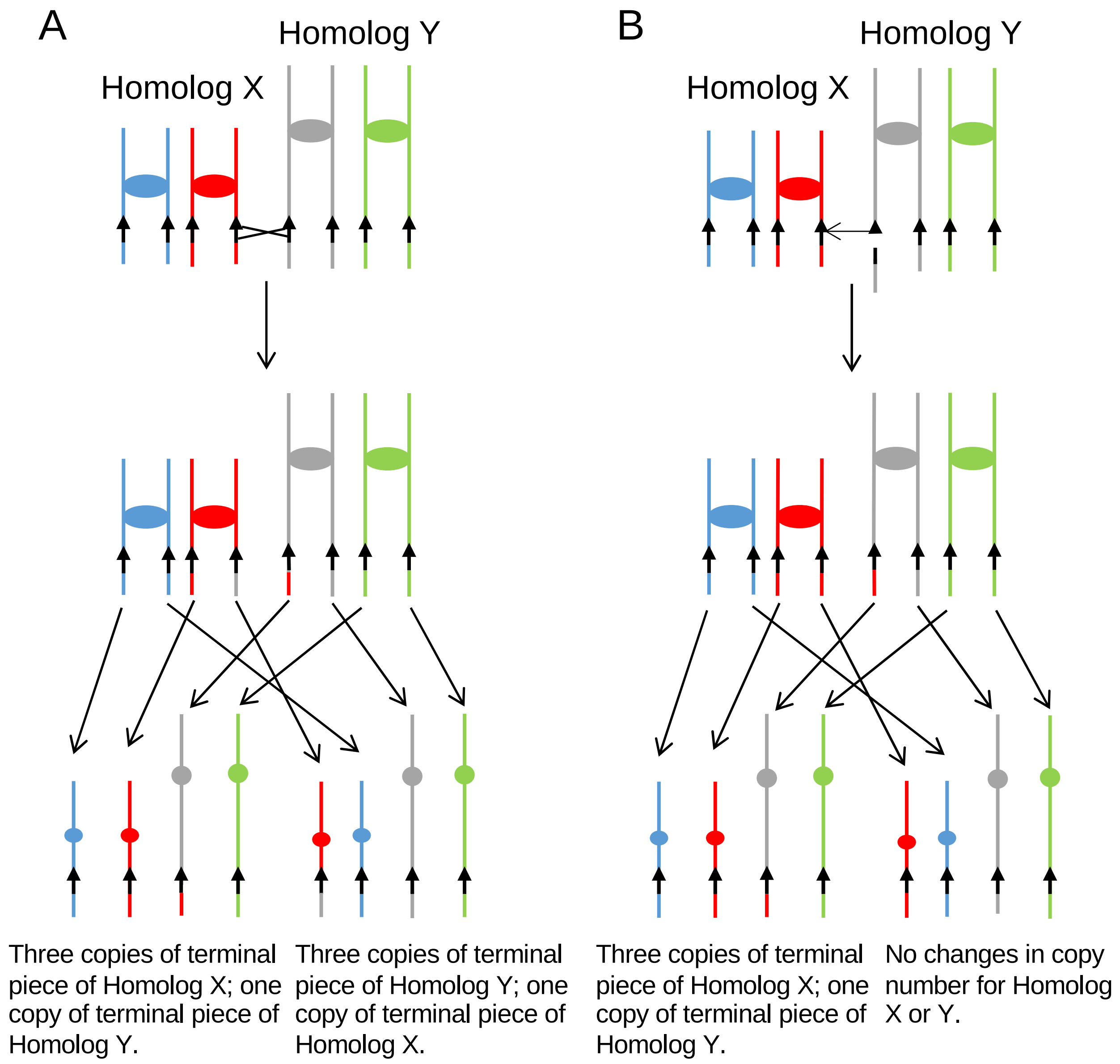

Figure S4. Schematic of translocation formation, and how it produces coupled deletions and duplications. In this figure, one pair of homologs (Homolog X) is shown by blue and red lines, and the second (Homolog Y) is shown by gray and green lines. The arrows show the positions of repeated elements on the chromosomes. Ovals/circles indicate the centromeres. **A.** Coupled deletions and duplications caused by a reciprocal crossover between repeats on non-homologous chromosomes. If the translocations segregate into different daughter cells, one would observe reciprocal patterns of coupled duplications and deletions. **B.** Coupled deletions and duplications caused by break-induced replication (BIR). In this mechanism, a break on the gray chromatid is repaired by invasion of the red chromatid at the repeat, followed by conservative replication of the red chromatid. Following chromosome segregation, one of the daughter cells would have a coupled deletion and duplication, whereas the other would not have a change in gene dosage.

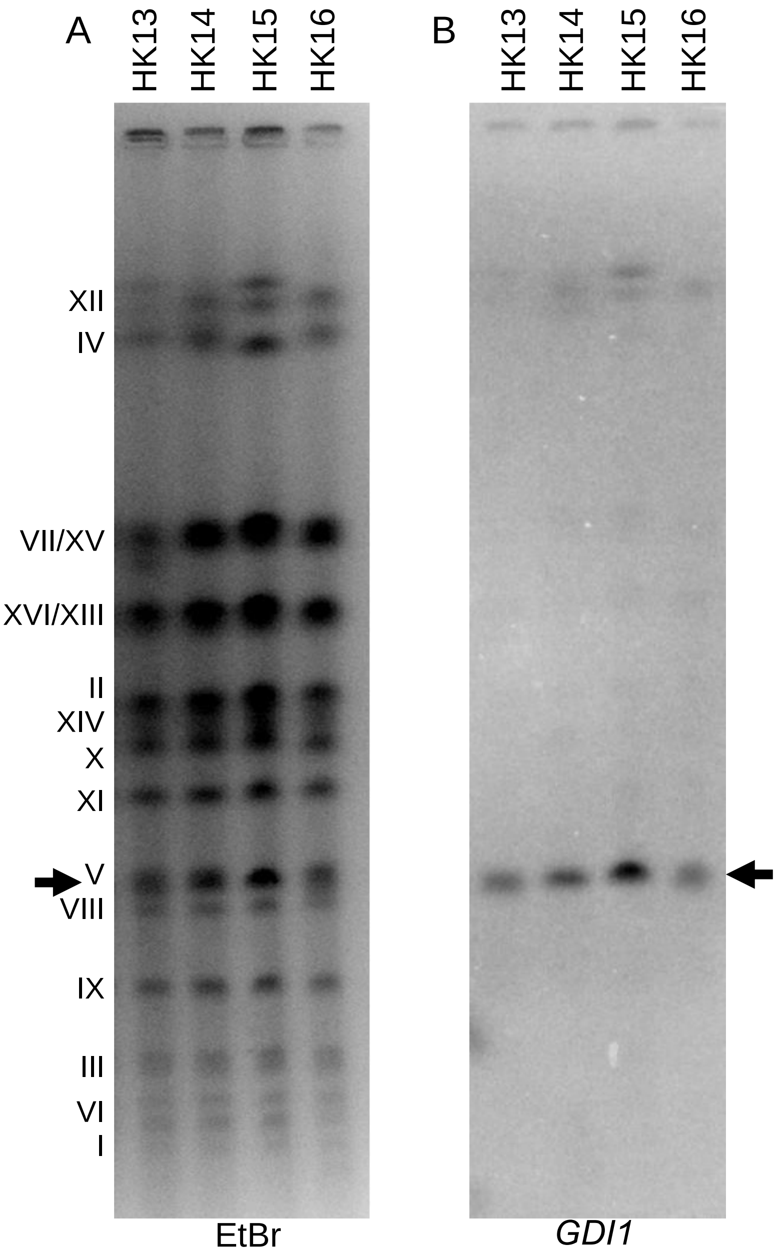

Figure S5. Translocation of XV and V (CHEF and Southern). Isolate HK14 has a pattern of terminal deletions and duplications that suggests a translocation of about 560 kb. The expected size of this translocation is about the same size as the intact chromosome V. As shown in Fig. S3B, HK14 has a chromosome of this size that hybridizes to a probe derived from chromosome XV. In this figure, we confirm that a probe from V (*GDI1*) hybridizes to a band of the appropriate size. Although this observation does not prove the existence of a translocation, it demonstrates that there are no novel bands that hybridize to the chromosome V probe. **A.** EtBr-stained CHEF gel used in the Southern analysis. **B.** Southern analysis with a chromosome V-specific probe.

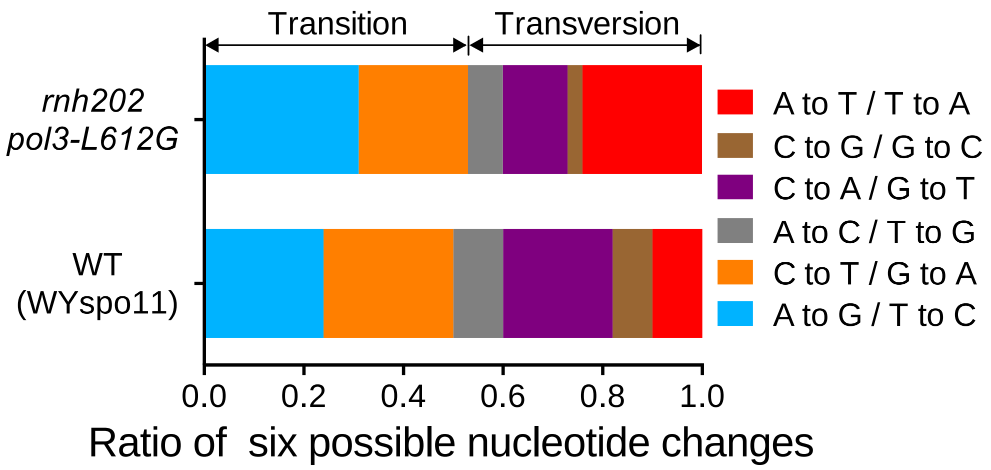

Figure S6. Distribution of single-base mutations of 6 types compared to wild-type. In this figure, we compare the pattern of single-base mutations observed in the wild-type strain (WYspo11; (5)) with the pattern observed in the *rnh202 pol3-L612G* strain. As discussed in the text, the numbers of mutations in each class in the two strains, compared by a 2x6 Fisher exact test, are very significantly different.

Dataset descriptions.

Dataset S1 (separate file). Analysis of LOH events, aneuploidy, and large deletion/duplications in ribodysgenic zygotes.

Dataset S2 (separate file). Association between LOH breakpoints and elements of chromosome structure in ribodysgenic hybrids.

Dataset S3 (separate file). Sequence of single-base mutations and small in/dels in ribodysgenic hybrids.

REFERENCES

1. Epshtein, A., Potenski, C. J., and Klein, H. L. (2016). Increased spontaneous recombination in RNase H2-deficient cells arises from multiple contiguous rNMPs and not from single rNMP residues incorporated by DNA polymerase epsilon. Microb. Cell, 3, 248-254.

2. Potenski, C.J., Niu, H., Sung, P. and Klein, H.L. (2014) Avoidance of ribonucleotide-induced mutations by RNase H2 and Srs2-Exo1 mechanisms. Nature, 511, 251-254.

3. Donnianni, R.A., Zhou, Z.X., Lujan, S.A., Al-Zain, A., Garcia, V., Glancy, E., Burkholder, A.B., Kunkel, T.A. and Symington, L.S. (2019) DNA polymerase delta synthesizes both strands during break-induced replication. Mol. Cell, 76, 371-381.

4. St Charles, J. and Petes, T.D. (2013) High-resolution mapping of spontaneous mitotic recombination hotspots on the 1.1 Mb arm of yeast chromosome IV. PLoS Genet., 9, e1003434.

5. Sui, Y., Qi, L., Wu, J.K., Wen, X.P., Tang, X.X., Ma, Z.J., Wu, X.C., Zhang, K., Kokoska, R.J., Zheng, D.Q. *et al.* (2020) Genome-wide mapping of spontaneous genetic alterations in diploid yeast cells. Proc. Natl. Acad. Sci. U.S.A., 117, 28191-28200.
